# Fecal leukocyte frequency in children with acute viral gastroenteritis: a single-center experience

**DOI:** 10.1590/1806-9282.20230972

**Published:** 2024-05-03

**Authors:** Dilek Konuksever, Sevinc Puren Yucel Karakaya

**Affiliations:** 1 Ankara Bilkent City Hospital, Pediatrics, Turkish Ministry of Health – Ankara, Turkey.; 2 Cukurova University, Department of Biostatistics – Adana, Turkey.

**Keywords:** Rotavirus, Adenovirus, Virus, Leukocyte, Children

## Abstract

**OBJECTIVE:**

Our objective was to determine the frequency of rotavirus, adenovirus, and rota-adenovirus co-infections and investigate the fecal leukocyte rate associated with these infections in patients with gastroenteritis.

**METHODS:**

This is a retrospective study. We identified patients who were admitted to the pediatric emergency department with acute gastroenteritis and had their stool samples tested for rotavirus and/or adenovirus antigens. Among them, we determined the individuals who underwent stool microscopy tests on the same day and recorded their results.

**RESULTS:**

A total of 1,577 patients who underwent testing for rotavirus and/or adenovirus antigens in their stool samples were identified. Among these patients, 583 individuals had concurrent fecal microscopy results. The prevalence of solely rotavirus antigen positivity was 16.4%, solely adenovirus antigen positivity was 2.9%, and rota-adenovirus co-infections were detected in 1.8% of the children. The fecal leukocyte rates in children infected with rotavirus, adenovirus, and rota-adenovirus co-infections were 4.8, 13.3, and 88.9%, respectively.

**CONCLUSION:**

The presence of fecal leukocytes was detected at a high rate in cases of viral gastroenteritis, especially in rota-adenovirus co-infections. Therefore, clinicians should not consider only bacterial pathogens in the presence of fecal leukocytes.

## INTRODUCTION

Acute gastroenteritis is a common disease affecting all age groups, especially children. It is mostly caused by viral agents^1,2^. Every year, viral gastroenteritis is the cause of death in more than 200,000 children worldwide^3^. Rotavirus and adenovirus are common causes of viral gastroenteritis in children^4^.

Rotavirus infection is confirmed as the important cause of severe diarrhea in children under 5 years of age. However, it affects all age groups worldwide. According to the reports, 45% of rotavirus deaths in 2016 occurred in people aged 5 years and older^5^. It is estimated that rotavirus caused the deaths of about 128,500 children under the age of 5 years and more than 258 million episodes of diarrhea worldwide in the same year^6^.

Rotavirus is known to cause noninflammatory and secretory, malabsorptive diarrhea by infecting intestinal villus enterocytes and enteroendocrine cells^2^. Despite widespread tissue damage and cell death in rotavirus infection, it is noteworthy that the inflammatory reaction is very limited. It has been reported that this may be due to the anti-inflammatory effects of rotavirus-induced cholinergic stimulation. Thus, tissue damage is also limited in this infection^7^.

Adenovirus is another important cause of gastroenteritis in children. It causes approximately 2–15% of acute diarrheal episodes in children^8^. Adenoviruses are subdivided into over a hundred types based on their biological and genetic characteristics. Adenovirus subgroups 40 and 41 are frequently responsible for gastroenteritis. The virus most commonly affects children younger than 2 years in low- and middle-income countries^9^.

Infectious diarrheas are classified as inflammatory and non-inflammatory according to their pathogenesis and clinical findings. One of the differences in this distinction is the presence of leukocytes in the stool accompanying inflammatory diarrhea^10^. Both rotavirus and adenovirus are non-invasive pathogens. Therefore, they are considered non-inflammatory agents of diarrhea. Nevertheless, they can interact with the enteric cells of the host to remove an inflammatory response so mild fecal leukocytes may be detected in feces^11^. However, there is limited data on the level of fecal leukocytes as inflammatory response markers in children infected with these agents.

The diarrhea management guidelines of the Infectious Diseases Society of America (IDSA)^12^ do not recommend fecal leukocyte tests for diagnosis and treatment planning. However, the detection of leukocytes in the fecal analysis still influences physicians’ decisions regarding antibiotic prescription^13^.

Therefore, this study was conducted to investigate the presence and quantity of fecal leukocytes in children infected with rotavirus, adenovirus, and co-infections of rotavirus and adenovirus. Additionally, the frequency of fecal leukocytes was compared among these viral agents. This study aimed to bring clinicians’ attention to the potential occurrence of fecal leukocytes in viral infections.

## METHODS

This study was approved by the local Ethics Committee in accordance with the Declaration of Helsinki (No. E-20/370).

We conducted a retrospective, cross-sectional study in the pediatric emergency department of a tertiary care hospital. We reviewed the medical records of children under the age of 18 years who were diagnosed with acute gastroenteritis between April 01, 2017, and December 31, 2020. Patients with rotavirus and adenovirus antigen (AdVA) test results on the same day were included in the study. Their age, gender, stool antigen test results, and application seasons were recorded.

In the second stage of the study, patients who underwent stool microscopy simultaneously with rotavirus and AdVA tests were identified and their results were recorded. Based on the results of stool antigen tests, the children were divided into four groups. Group 1: negative for both rotavirus antigen (RVA) and AdVA, group 2: negative for RVA and positive for AdV, group 3: positive for RVA and negative for AdVA, and group 4: positive for both RVA and AdVA.

The presence of rotavirus and AdVAs in fresh stool samples was investigated using a commercial immunochromatographic test kit (AV-RV Combo test, Rapid Diagnostic Test, Turkey). The test kit utilized a nitrocellulose membrane coated with monoclonal anti-rotavirus and anti-adenovirus antibodies as the scanning element. The test method is a rapid, qualitative approach based on the principle of the formation of antigen-antibody complexes on the test region (T1–T2) of the test card, resulting in a visible pink line after the binding of RVA and/or AdVA to the antibody-coated membrane following a 10–15-min incubation period, according to the manufacturer's recommendations. The presence of ≥5/mL leukocytes in stool samples was accepted as a sign of inflammatory diarrhea^14^.

### Statistical analysis

Categorical variables were expressed as numbers and percentages. The chi-square test for categorical variables was used to compare the distributions of age, gender, and season characteristics between the rotavirus and AdVA positivity groups. The chi-square test was also used to compare the distributions of age, gender, and season in the rotavirus, adenovirus, and coinfection antigen positivity groups. Here, the Bonferroni method was used to compare column proportions, adjusted p-value were obtained, and the findings are presented with letters in the relevant table. All analyses were performed using the IBM SPSS Statistics Version 20.0 statistical software package. All tests were two-sided, and the statistical level of significance for all tests was considered to be 0.05.

## RESULTS

A total of 1,577 cases were included in the study. RVA positivity was detected in 288 (18.3%) patients, and AdVA positivity was detected in 74 (4.7%) patients. Gender, age, and seasonal distribution according to adenovirus and rotavirus positivity are given in Table 1. Adenovirus positivity is higher in females (p=0.003), but it does not show a statistically significant difference according to age and season (p=0.884 and p=0.624, respectively). Rotavirus positivity did not differ according to gender (p=0.192), but it showed statistically significant differences according to age and season (both p<0.001). While rotavirus positivity is high in the 13–24-month age group, it is low in children older than 12 years. In addition, the rotavirus positivity is higher in spring, followed by winter, autumn, and summer.

**Table 1:** Distribution of characteristics by adenovirus and rotavirus antigen positivity.

Characteristics		Adenovirus		p	Rotavirus		p
		Negative	Positive		Negative	Positive	
		n (%)	n (%)		n (%)	n (%)	
Gender	Female	630 (41.9)	44 (59.5)	0.003	541 (42.0)	133 (46.2)	0.192
	Male	873 (58.1)	30 (40.5)		748 (58.0)	155 (53.8)	
Age	0–12 months	113 (7.5)	5 (6.8)	0.884	97 (7.5)	21 (7.3)	<0.001
	13–24 months	621 (41.3)	29 (39.2)		498 (38.6)	152 (52.8)	
	25–72 months	284 (18.9)	14 (18.9)		243 (18.9)	55 (19.1)	
	6–12 years	333 (22.2)	20 (27.0)		304 (23.6)	49 (17.0)	
	13–18 years	152 (10.1)	6 (8.1)		147 (11.45)	11 (3.8)	
Season	Spring	387 (25.7)	15 (20.3)	0.624	265 (20.6)	137 (47.6)	<0.001
	Summer	471 (31.3)	23 (31.1)		465 (36.1)	29 (10.1)	
	Autumn	380 (25.3)	23 (31.1)		357 (27.7)	46 (16.0)	
	Winter	265 (17.6)	13 (71.6)		202 (15.7)	76 (26.4)	
Total		1503 (95.3)	74 (4.7)		1289 (81.7)	288 (18.3)	

In our study, only adenovirus or rotavirus positivity and both antigen positivity and negativity were also evaluated. Only RVA positivity was detected in 259 (16.4%), only AdVA positivity was detected in 45 (2.9%), and rota-adenovirus co-infections were detected in 29 (1.8%) of the children. The distribution of characteristics according to these groups is given in Table 2.

**Table 2:** Distribution of characteristics according to rotavirus, adenovirus, and coinfection antigen positivity.

Characteristics		Group				p
		RVA-/–/AdV-–	RVA-/–/AdV+	RVA+/AdV-–	RVA+/AdV+	
		(n=1244)	(n=45)	(n=259)	(n=29)	
Gender, n (%)						
	Female	514 (41.3)^a^	27 (60.0)^b^	126 (45.3)^a^	17 (58.6)^b^	0.019
	Male	730 (58.7)^a^	18 (40.0)^b^	152 (54.7)^a^	12 (41.4)^b^	
Age, n (%)						
	0-–12 months	96 (7.7)^a^	1 (2.2)^a^	17 (6.6)^a^	4 (13.8)^a^	<0.001
	13-–24 months	471 (37.9)^a^	27 (60.0)^b^	150 (57.9)^b^	2 (6.9)^c^	
	25-–72 months	231 (18.6)^a^	12 (26.7)^a^	53 (20.5)^a^	2 (6.9)^a^	
	6-–12 years	299 (24.0)^a^	5 (11.1)^a,b^	34 (13.1)^b^	15 (51.7)^c^	
	13-–18 years	147 (11.8)^a,b^	0 (0.0)^b,c^	5 (1.9)^c^	6 (20.7)^a^	
Season, n (%)						
	Spring	261 (21.0)^a,b^	4 (8.9)^b^	126 (48.6)^c^	11 (37.9)^a,c^	<0.001
	Summer	447 (35.9)^a^	18 (40.0)^a^	24 (9.3)^b^	5 (17.2)^a,b^	
	Autumn	340 (27.3)^a^	17 (37.8)^a^	40 (15.4)^b^	6 (20.7)^a,b^	
	Winter	196 (15.8)^a^	6 (13.3)^a,b^	69 (26.6)^b^	7 (24.1)^a,b^	

Each subscript letter denotes a subset of groups whose column proportions do not differ significantly from each other at the 0.05 level.

In addition, stool microscopy evaluation was also performed in 583 (36.9%) of these patients, and the distribution of erythrocytes and leukocytes is summarized in Table 3. Fecal leukocyte rates in children infected with rotavirus, adenovirus, and rota-adenovirus co-infections were 4.8, 13.3, and 88.9%, respectively.

**Table 3:** Erythrocyte and leukocyte distribution in patients with stool microscopy evaluation.

		RVA–/AdVA–	RVA–/AdVA+	RVA+/AdVA–	RVA+/AdVA+	p
		(n=475)	(n=15)	(n=84)	(n=9)	
Erythrocyte, n (%)						
	No	425 (89.5)^a^	15 (100.0)^a^	81 (96.4)^a^	3 (33.3)^b^	<0.001
	<5	19 (4.0)^a^	–^a,b^	1 (1.2)^a^	2 (22.2)^b^	
	>5	31 (6.5)^a^	–^a^	2 (2.4)^a^	4 (44.4)^b^	
Leukocyte, n (%)						
	No	324 (68.2)^a^	12 (80.0)^a,b^	75 (89.3)^b^	1 (11.1)^c^	<0.001
	<5	43 (9.1)^a^	1 (6.7)^a^	5 (6.0)^a^	–^a^	
	>5	108 (22.7)^a^	2 (13.3)^a,b^	4 (4.8)^b^	8 (88.9)^c^	

AdVA: adenovirus antigen; RVA: rotavirus antigen. Each subscript letter denotes a subset of groups whose column proportions do not differ significantly from each other at the 0.05 level.

## DISCUSSION

In this study, we compared the fecal leukocyte rates in children diagnosed with acute gastroenteritis in the pediatric emergency clinic who had rotavirus, adenovirus, and rota-adenovirus co-infections as part of the disease etiology. The detection rates of rotavirus, adenovirus, and rota-adenovirus coinfections in children with acute gastroenteritis for whom antigen testing was requested were 16.4, 2.9, and 1.8%, respectively. Fecal leukocyte detection rates were determined most frequently in rota-adenovirus coinfections and least in rotavirus infections, inversely proportional to the frequency of gastroenteritis agents.

In line with previous studies, we also observed a higher prevalence of rotavirus compared with adenovirus in patients with acute gastroenteritis in our study^1,15,16^. The frequency of acute gastroenteritis caused by rotavirus and adenovirus varies across different countries. We believe that this variability may be attributed to differences in detection methods, the age distribution of the tested populations, hygiene and sanitation conditions, and the inclusion or exclusion of rotavirus in the vaccination schedule.

In a study conducted in Turkey, the frequency of rotavirus, adenovirus, and rota-adenovirus co-infection was found to be 12.6, 2.6, and 0.14%, respectively^4^. These results were lower than ours. We think that this is because the age group in this study covers the 0–76 years age group. However, some studies report that the frequency of rotavirus and adenovirus is lower than this study, although it includes younger age groups^17^. We suppose that it may be related to hygiene conditions or vaccination. In the other study, rotavirus, adenovirus, and rota-adenovirus co-infection frequencies were detected at 22, 10.3, and 1.1%, respectively. This study was conducted with children under 5 years of age with acute gastroenteritis^18^. We think that the more frequent detection of viral antigen positivity compared with our study is due to the greater susceptibility to viral infections in this age group. However, the frequency of co-infection rates was higher in our study. However, when we evaluated the frequency of co-infection rates under 6 years of age, we detected the frequency as 0.5% in our study, which was lower compared with the other study.

Viral gastroenteritis can lead to a wide range of clinical conditions, ranging from asymptomatic infections to severe dehydration. Clinically differentiating between viral and bacterial etiology in patients with gastroenteritis is not always possible. However, the treatment plan varies depending on the viral or bacterial etiology. For instance, in cases of invasive gastroenteritis infections caused by bacteria, appropriate antibiotic therapy is crucial to minimize mortality and morbidity. Stool cultures are commonly utilized to identify bacterial agents. However, this method typically yields results after several days and has limited sensitivity in detecting bacterial pathogens. One of the debated methods for identifying intestinal inflammation in infectious gastroenteritis is the presence of fecal leukocytes. Fecal leukocytes can increase enteroinvasive gastrointestinal infections. However, fecal leucocytes may be observed in some cases of viral diarrhea^19^. In our study, fecal leukocyte rates detected in children infected with rotavirus, adenovirus, and rota-adenovirus co-infections were 4.8, 13.3, and 88.9%, respectively. There was a remarkable increase in fecal leukocyte rates in rota-adenovirus co-infections. In a study, it was reported that the frequency of rotavirus was 10.7%, adenovirus was 5%, and rota-adenovirus co-infections were 1.4% among children under 10 years of age. In the same study, the frequency of fecal leukocytes was detected at 3.3% in patients with only rotavirus, 22% in those with only adenovirus, and 48.4% in those with co-infection with rota-adenovirus^20^. In this study, similar to our findings, fecal leukocytes were most frequently detected in rota-adenovirus coinfections and least frequently in rotavirus infections.

The mechanisms underlying viral coinfection have not yet been fully elucidated^21^. We propose a hypothesis that rotavirus infections may exhibit specific mechanisms that increase the likelihood of secondary infections, as supported by the observed higher frequency of coinfections during periods of elevated rotavirus prevalence. When we evaluated the seasonal variability for the caused agent, rotavirus positivity was found most frequently in the spring, adenovirus positivity in the autumn, and rota-adenovirus co-infection most often in the spring. Xiao et al., found that rotavirus positivity was most common in winter, adenovirus in summer, and rota-adenovirus co-infection most commonly in winter. In both Xiao's^21^ study and our own, the season characterized by a high prevalence of rota-adenovirus coinfection coincided with the season when rotavirus was frequently detected.

Zaraket et al., investigated the frequencies of rotavirus, adenovirus, and rota-adenovirus co-infections and found them to be 17, 6.5, and 2.1%, respectively. Furthermore, they observed elevated levels of inflammatory markers such as white blood cells, absolute neutrophil count, and C-reactive protein in the co-infection group compared with the groups with rotavirus or adenovirus alone^15^. In another study, the detection rates of fecal leukocytes in gastroenteritis were found to be 19% in the bacterial pathogens and 7.9% in the virus agents, and this difference was not found to be significant in detecting bacterial and virus differentiation in the etiology of gastroenteritis^22^.

This study has several limitations. First, it is not possible to definitively exclude bacterial infections in children diagnosed with inflammatory diarrhea due to the lack of stool culture results. Second, as is known, the presence of leukocytes in feces may be also an indicator of antibiotic-associated colitis, pseudomembranous colitis, and idiopathic inflammatory bowel diseases. Although patient files are examined in detail in terms of these diseases, we cannot be sure that they have been completely ruled out because our study is retrospective. Third, this study is single-centered, so its generalizability is low.

## CONCLUSION

This study showed that viruses, especially rota-adenovirus co-infections, might play a role in inflammatory diarrhea. Therefore, antimicrobials should not be routinely recommended when fecal leukocytes are detected in patients with gastroenteritis.
